# Theatre and laboratory workers’ awareness of and safety practices against hepatitis B and C infection in a suburban university teaching hospital in Nigeria

**Published:** 2012-09-02

**Authors:** Emmanuel Chidiebere Okwara, Oguamanam Okezie Enwere, Chiekulie Kevin Diwe, Jerome Emeka Azike, Alexander Emeka Chukwulebe

**Affiliations:** 1 Department of Chemical Pathology, Imo State University Teaching Hospital, Orlu, Imo State, Nigeria; 2 Department of Internal Medicine, College of Medicine, Imo State University, Orlu Campus, Imo State, Nigeria; 3 Department of Community Medicine, College of Medicine, Imo State University, Orlu Campus, Imo State, Nigeria; 4 Department of Surgery, College of Medicine, Imo State University, Orlu Campus, Imo State, Nigeria

**Keywords:** HIV, HCV, HBV vaccination, healthcare workers, safety measures

## Abstract

**Introduction:**

The consistent use of barrier protection among theatre workers is low in this region, so also is hepatitis B virus (HBV) vaccination. We assessed the level of awareness of HBV and hepatitis C virus (HCV), HBV vaccination and adoption of safety measures by theatre and laboratory workers.

**Methods:**

Structured questionnaires were administered to these workers which assessed level of knowledge of the viruses, practice of barrier protection and level of HBV vaccination.

**Results:**

Of 169 participants 32.5% were laboratory workers, 67.5% were theatre workers; 29.6% males, 70.4% females. Most 94% (159) were aware that HBV and HCV are viral infections, while 77% (127) and 72.1% (119) knew HBV and HCV are transmitted through blood transfusion and needle stick injuries; a correct knowledge was significantly better among respondents with tertiary education (OR 2.7; 95%CI 1.2-6.3 and OR 2.3; 95%CI 1.0-5.1 respectively). Although 49.1% (80) were aware unprotected sex was a route of transmission, laboratory staff was twice as likely to have this knowledge (OR 2.1; 95% CI 1.08-4.08). Only 67.5% (114) use safety measures consistently, while 86 (54.8%) had received the vaccine of which only 48 (29.78% of total respondents) had completed three (3) doses; more likely among those with tertiary education (OR 2.6; 95%CI 1.2-5.8).

**Conclusion:**

Most (94%) workers were aware of the risk of HBV and HCV and HBV vaccine (92.9%) but only few (29.78%) completed vaccination. Unfortunately, only 2/3 use protective measures consistently. There is need to make vaccination of health care workers against HBV infection a firm policy and ensure complete and consistent adherence to work standard safety measures.

## Introduction

Hepatitis B virus (HBV) infection and hepatitis C virus (HCV) infection are among the commonest occupational risks healthcare workers are exposed [1]. The infections are acquired in the hospital setting via needle prick injuries from contaminated needles, eye contact of infected body fluids or from contact of infected body fluids with broken skin. Among the health care workers, theatre and laboratory staffs are at a risk of HBV and HCV infection from their contact with infected materials and patients [1]. Among a Nigerian population, prevalence of HBV was 6% [2] with the highest infectious rate observed among those aged 21-30 years, in Pakistan the prevalence of HBV and HCV infection among health care workers was 6.0% and 5.4% respectively [3]. In Uganda 8.1% of health care workers were seropositive to HBV with 67.8% prevalence of needle stick injuries and 41.0% prevalence of exposures to mucous membranes such as the eyes [4] whereas in Pakistan 2.18% of health care workers were seropositive to HBV, nurses and technicians being more prone to occupational exposure to HBV [5]. The use of gloves have been demonstrated to reduce infection, however their use among healthcare workers is inconsistent and may be influenced by risk perception and health care culture [5]. Use of eye shields/goggles by theatre workers is even more inconsistent.

Preparation of patients for operative procedures in Eastern Nigeria does not routinely include screening for HBV and HCV but may include screening for human immunodeficiency virus (HIV). Theatre workers may often be unguarded when attending to patients who are negative to HIV. This places the theatre worker at a higher risk of HBV and HCV infection. Therefore, the adoption of universal and consistent safety practices is important in reducing these occupational infections.

We evaluated the awareness of and safety practices against HBV and HCV infection as well as HBV vaccination amongst theatre and laboratory staff, and the impact of their level of education, if any, on their safety practice in a Nigerian University Teaching Hospital with a view to suggesting policy related changes, if necessary, towards improving safety practices.

## Methods

This study was a prospective study conducted at the Imo State University Teaching Hospital, Orlu, Imo State, South-eastern Nigeria, a tertiary care hospital located thirty (30) kilometers from the state capital, Owerri. Orlu is a semi-urban town with an estimated population of 220,000. The hospital is a 200-bed teaching hospital serving as a training center for undergraduate medical students, resident doctors and nursing students.

The study population was all laboratory and theatre staff of the hospital who gave their oral consent to participate in the study. A 29-item structured self-administered questionnaire was given to all laboratory and theatre staff who accented to participate in the study. Cleaners, porters and laboratory assistants had their questionnaires interviewer-administered due to their limitation of understanding in the technical terms used. The identity of participants was protected as participants’ names were not required. Completed questionnaires were retrieved immediately by the study investigators. Workers who were on work leave or who declined to participate in the study were excluded.

One hundred and sixty nine (169) questionnaires were administered. The data collected was entered into a password protected computer and analyzed using SPSS version 16. In the statistical analysis, frequencies, mean values and percentages were presented. Odds ratio and chi square were used to compare variables. A chi square P value < 0.05 was considered statistically significant.

## Results

There were 169 workers who participated with an average age of 35.4years (±8.6) and a median period of practice of 7years (range 1-35years); 32.5% (55) were laboratory workers while 67.5% (114) were theatre workers; also 29.6% (50) were males and 70.4% (119) were females. Medical doctors comprised 7.7% (13), 43.2% (73) were nurses and 11.8% (20) were medical laboratory scientists. Others include medical laboratory technicians 8.9% (15), medical laboratory assistants 10.1% (17) and cleaners 14.2% (24). Majority of respondents had tertiary level education 79.9% (135) while the rest had only secondary education or less 20.1% (34). Also 69.2% (117) were married.

## Knowledge

Most respondents 94% (159) were aware that HBV and HCV are viral infections, while 6% (10) didn't recognize they are viral infections. While 77% (127) and 72.1% (119) know HBV and HCV can be transmitted through blood transfusion and needle stick injuries, a correct knowledge was significantly better among respondents with tertiary education compared to others (OR 2.7; 95%CI 1.2-6.3 and OR 2.3; 95%CI 1.0-5.1 respectively). However knowledge of above risk was similar in both departments (p= 0.79 and 0.8 respectively). Only 49.1% (80) were aware unprotected sex was a route of transmission which was unrelated to level of education (p= 0.17). But laboratory staff were twice as likely to believe the viruses could be sexually transmitted (OR 2.1; 95% CI 1.08-4.08).

Smaller proportions (41.2% theatre, and 39% laboratory) were aware the viruses could be transmitted through contact of the eyes or broken skin with infected body fluids irrespective of education (p= 0.34 and 0.84 respectively).

Majority of the respondents (95.9% and 92.9% respectively) believe that HBV and HCV can be contacted at work and were aware of the existence of the HBV vaccine.

## Practice

Most respondents (89.3%) admitted using any safety measure but only 67.5% (114) reportedly use these safety measures consistently. Of those who applied any safety measures, 43.8% use surgical gloves alone, 18.3% use gloves, aprons and boots while 21.9% use all of gloves, aprons, boots and eye shields while 8.4% use either of aprons, boots or eye shields.

Of the 157 (92.9%) respondents who were aware of the existence of the HBV vaccine, 86 (54.8%) had received the vaccine of which only 48 (55.8%) or 29.78% of all respondents had completed three (3) doses. Those with tertiary education were significantly more likely to have had the HBV vaccine (OR 2.6; 95%CI 1.2-5.8), with a higher proportion having received three (3) doses (OR 2.1; 95%CI 0.6-8.0) compared to those with less than tertiary education. Also theatre staffs were significantly more likely to have received the vaccine compared to laboratory staff (OR 2.9; 95%CI 1.5-5.7) but there is little difference in the proportion of those who had had three (3) doses in both departments (OR 1.3; 95%CI 0.5-3.7).

**Figure 1:**
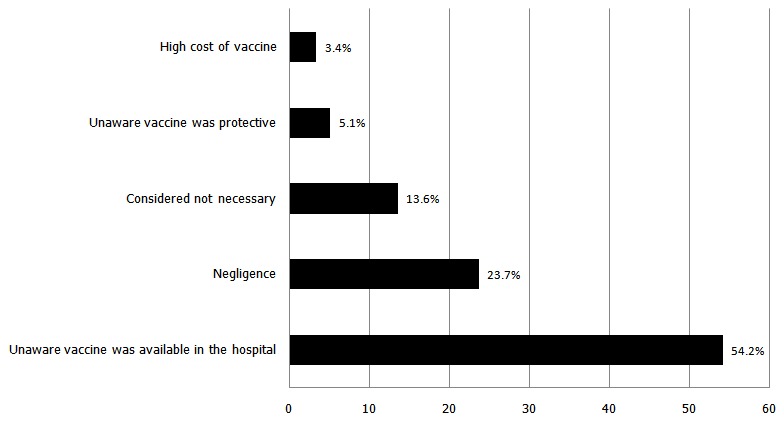
Reasons for not receiving HBV vaccine (N=71)

The 71 health workers aware of the vaccine but who had not received any dose of HBV vaccine gave different reasons for not obtaining the vaccine (Figure 1). Only 35.2% (58) of the respondents had pre-employment screening for HBV and HCV.

## Discussion

Health workers may be at higher risk of contracting HBV and HCV infections than the general population. These viruses are 50 to 100 times more infectious than HIV [6]. The theatre worker may not be adequately guarded while attending to patients who are negative to HIV. Even though these patients are negative for HIV, their HBV and/or HCV is unknown to the health care worker if no screening tests have been conducted. Although HBV infection is similar in Nigerian [2] and Pakistan [3] populations with a higher preponderance among males, it is slightly lower in a Chinese population with 4.38% also seropositive to HBV [7]. In another study in Nigeria 17.1% of blood donors were seropositive to HBV [8] while 34.9% of patients with chronic liver disease were seropositive in Pakistan [9]. These high proportions among the general population may suggest that the health care worker may be unknowingly exposed to the risk of HBV infection while at work. Theatre and laboratory workers are most times in contact with blood and other tissue fluids making them at high risk of contacting HBV infection. The need for the adoption of appropriate and consistent safety measures by the health care worker is paramount in view of the prevalence of HBV infection in the general population and the high frequency of potentially contaminating accidents among health care workers [4, 10].

In this study we observed that most respondents were aware that HBV and HCV were viral infections; however the knowledge of the route of transmission was better among workers with higher education irrespective of their departments. It is likely that this group of workers have had general education on the nature of these organisms through their training. However, knowledge of unprotected sexual intercourse as a route of viral transmission was unrelated to level of education but better among laboratory workers. Since the activities of our respondents involve handling body tissue and fluids, it is of great concern that less than half our respondents believe the viruses can be transmitted though eye contact and skin abrasions with infected materials and only about a third use protective measures consistently even when they are available for their use.

Single dose HBV vaccine gave protective antibodies only to 6.3% of a Nigerian population, while 98.3% developed protective antibodies after the second dose [11]. Three doses of HBV vaccination provide 100% protection against HBV infection [11]. Despite the knowledge of HBV infection and HBV vaccine the level of vaccination among the respondents is unacceptably low with only less than a third of the study population having completed HBV vaccination. It was also not surprising to note that respondents with a better education were more likely to have knowledge of the vaccine with a higher proportion having completed the vaccination regimen. Only 37.5% of Residents Doctors in Paediatrics at a Brazilian tertiary hospital received 3 doses of HBV vaccine [10]. HBV vaccine in the study hospital is available free-of-cost yet 54.2% of our study population who had not been immunized were not aware the vaccine was available in the hospital while 3.4% attributed non immunization to cost of vaccine. In Pakistan, barriers to complete vaccination in spite of good knowledge of subject were negligence (38.8%), work pressure (39.8%), high cost of vaccine (20.9%) and unavailable vaccine (0.5%) [4].

**Limitations** We did not establish the HBV or HCV status of our respondents as this is a medically sensitive and personal information. A positive test to HBV is a contraindication for HBV vaccination. The number involved is small. However we recognize that the scope is limited to a section of hospital workers most prone to accidental HBV or HCV transmission. This is with a view to addressing any observed deficiencies noted to ensure better protection of our hospital workers.

## Conclusion

Most (95.9%) theatre and laboratory workers were aware of occupational risk of HBV and HCV infections and also aware of HBV vaccine (95.2 5%) but only few (29.78%) received complete HBV vaccination. Unfortunately, only about 2/3 use protective measures consistently. There is need to make vaccination of health care workers against HBV infection a policy as part of the employment process and ensure complete and consistent adherence to standard safety measures against HBV and HCV infection in the workplace.
